# Therapeutic validity and effectiveness of exercise interventions after lower limb-salvage surgery for sarcoma: a systematic review

**DOI:** 10.1186/s12891-023-06315-y

**Published:** 2023-03-23

**Authors:** H. W. van Kouswijk, H. G. van Keeken, J. J. W. Ploegmakers, G. H. Seeber, I. van den Akker-Scheek

**Affiliations:** 1 Department of Orthopaedics, University of Groningen, University Medical Center Groningen, Groningen, The Netherlands; 2 Center for Human Movement Sciences, University of Groningen, University Medical Center Groningen, Groningen, The Netherlands; 3 University Hospital for Orthopaedics and Trauma Surgery Pius-Hospital, Medical Campus University of Oldenburg, Oldenburg, Germany

**Keywords:** Systematic review, Therapeutic validity, Exercise therapy, Bone neoplasms, Lower extremity

## Abstract

**Background:**

An increasing number of patients are surviving sarcoma after lower limb-salvage surgery (LSS) and are left with functional limitations. This systematic review aimed to determine the therapeutic validity and effectiveness of exercise interventions after lower limb-salvage surgery (LSS) for sarcoma.

**Methods:**

A systematic review was conducted using formal narrative synthesis of intervention studies (with and without control group) identified through PubMed, Embase, Cochrane Library, CINAHL, and PEDro databases. Studies were included if participants were treated with LSS for unilateral lower limb sarcoma and followed an exercise intervention using active exercise, physical training, or rehabilitation before and/or after surgery. This review’s outcome measures were interventions’ therapeutic validity, assessed using the CONTENT scale (0 to 9); methodological quality, identified using the Downs & Black checklist (0 to 28); interventions’ effectiveness, assessed based on differences in outcome measures between intervention and control groups; and certainty of evidence, classified according to the GRADE approach.

**Results:**

Seven studies involving 214 participants were included. None of the included interventions were therapeutically valid (median 5, range 1–5). All but one study were of at least fair methodological quality (median 18, range 14–21). There was very low-quality evidence that exercise interventions resulted in increased knee range of motion (MD 10–15°) or compliance (MD 30%), and reduced functionality scores (MD -5%) compared to usual care.

**Conclusions:**

We found overall low therapeutic validity of interventions, performed in overall low-quality studies. Combined with the very low certainty of evidence, the results prevent drawing valid conclusions on the interventions’ effectiveness. Future studies should aim for uniformity among their methodological approaches and outcome measures, using the CONTENT scale as a template to avert insufficient reporting.

**Trial registration:**

PROSPERO CRD42021244635.

**Supplementary Information:**

The online version contains supplementary material available at 10.1186/s12891-023-06315-y.

## Background

About 1% of all cancer diagnoses in Europe consist of malignant bone and soft tissue tumours, mainly different types of sarcoma [1]. About 0.5–2.0 per 100,000 individuals are diagnosed with sarcoma each year worldwide [1]. Thanks to the development of novel surgical procedures and therapeutic measures, the number of sarcoma survivors has significantly increased in Europe since 2005 [2], after having hardly improved since the 1980s [3–6]. Especially in the Netherlands, the survival rate of patients with sarcoma has improved, from 61% in 1999–2001 to 72% in 2005–2007 [2].

Sarcoma management follows a multidisciplinary approach in which both surgery and rehabilitation play an important role [7]. In the past decades, novel extremity-salvaging surgical procedures became available as alternatives to amputation [8]. Limb-salvage surgery (LSS) consists of complete excision of the lesion with clear margins, followed by bone or joint reconstruction using endoprostheses, among others. These interventions aim for disease-free survival while maintaining maximum function. Improved life expectancies and surgical innovations have increased survivors’ need to achieve and maintain optimal functionality and return to normal life. Evidence shows exercise therapy (as well as psychological acceptance) may be helpful during this process [9–12].

Exercise therapy can be defined as planned, structured, and repetitive activity aiming to improve physical performance [13]. It is characterised by specified criteria such as frequency, intensity, and type (e.g., strengthening, endurance, and functional exercise [14]). When applied in rehabilitation, exercise has positive effects on many diseases, including musculoskeletal disorders and cancer [15]. Exercise affects functional impairments through improved balance, muscle strength, and endurance, relieving cancer-related fatigue and strengthening physical ability [15, 16]. It is thought to boost patients’ self-confidence and psychological well-being [15]. Despite established benefits in several patient populations, prescribing exercise remains challenging in patients with sarcoma.

In sarcoma rehabilitation, clinicians often perceive exercise as a contraindication due to concerns about aggravating skeletal-related events [17]. However, keeping the International Classification of Functioning, Disability and Health (ICF) model [18] in mind, regaining preoperative functional levels is crucial to patients’ resumption of activities of daily living (ADLs), participation in home and community activities, and return to work and physical activity or sports [19]. Yet, patients continue to experience functional limitations after lower LSS [12]. As exercise has shown positive effects in related patient populations [15], it is worth exploring the effects of exercise in patients after lower LSS for sarcoma.

A persistent problem in determining exercise interventions’ effect is the heterogeneity of training programmes from the literature [20]. Therefore, it is recommended that interventions’ therapeutic validity be examined in systematic reviews [20, 21]. Accordingly, the Consensus on Therapeutic Exercise Training (CONTENT) scale has been developed, where therapeutic validity is defined as “the potential effectiveness of a specific intervention given to a potential target group of patients” [21]. This scale has been used before in systematic reviews on exercise interventions following, among others, joint replacement [14, 21] and intra-abdominal cancer surgery [22], revealing overall low therapeutic validity (score < 6 out of 9). As no such review exists for interventions after surgical treatment of sarcoma in the lower extremities, this systematic review aimed to answer the following questions:

1. What is the therapeutic validity of exercise interventions after lower LSS for sarcoma?
2. What is the exercise interventions’ effectiveness?

## Methods

The protocol for this systematic review was published in the PROSPERO database [23]. All reporting followed the Preferred Reporting Items for Systematic reviews and Meta-Analyses (PRISMA) guidelines [24].

### Identification and selection of studies

PubMed, Embase, Cochrane Library, CINAHL, and PEDro databases were searched from inception up to 9 February 2023 to retrieve eligible articles. The search strategy (see Additional file 1) was optimised for each database by an experienced scientific librarian of University Medical Center Groningen. Reference lists of the included articles were screened manually for additional relevant references by one reviewer (HWvK).

Identified articles’ eligibility was assessed by two researchers (HWvK & GHS) who were blinded from each other’s assessments, using the systematic review managing software Covidence (Veritas Health Innovation, Melbourne, Australia). The first screening was based on title and abstract, which were examined for eligibility against pre-defined criteria (Table 1). Articles not definitely excluded by this screening were obtained in full-text for further assessment. Eligible studies underwent data extraction. Disagreements were solved in a consensus meeting. A third researcher (IvdAS) was consulted if disagreement between individual judgements persisted.

**Table 1:** Inclusion criteria

Design:
- Randomised and clinical controlled trials
- Cross-sectional, cohort, and case–control studies
- Case-series
Participants:
- N ≥ 10
- Mean/median age ≥ 18
- Underwent lower limb-salvage surgery for any type of bone cancer
- Unilateral, primary surgery
Intervention:
- Any type of pre- or postoperative exercise intervention, without any further restrictions, as long as the movements were performed actively by the patient
- Less than six months between surgery and the intervention’s start
Outcome measures:
- Focussing on physical activity, physical activity behaviour and/or functioning, or mental health
Control group (not mandatory):
- Healthy controls, usual care, or another exercise intervention

### Characteristics of included studies

#### Methodological quality

The methodological quality of both randomised and non-randomised studies was assessed using an adjusted version of the ‘Checklist for Measuring Quality’ by Downs & Black [25]. This original scale consists of 27 questions on quality of reporting, external validity, bias, confounding, and statistical power. Answers were ‘yes’, ‘no’, or ‘unable to determine’, resulting in a score of 1 or 0 points. There is one exception, the Reporting subscale, which scored 0 to 2. In accordance with previous systematic reviews [26, 27], it was decided to modify the original Power item (dealing with sufficient statistical power) as the question was deemed unclear and could not be adequately resolved. This item was simplified to a score of 0 (no sample size calculation) or 1 (sample size calculation reported). The checklist’s total score was the sum of the items’ scores and therefore ranged from 0 to 28. A total score ≤ 14 indicates poor, 15–19 fair, 20–25 good, and 26–28 excellent methodological quality [28].

#### Data collection

The following data were collected to evaluate clinical heterogeneity between studies: bibliometric data (authors, country, publication year), study start and end dates, study design, sample size, population description (age, female percentage, BMI), tumour morphology (e.g., osteosarcoma, Ewing sarcoma, chondrosarcoma, primary or metastatic), tumour location, type of surgical procedure, resection amplitude, complications, type and characteristics of exercise interventions (intensity, session duration, frequency, setting, supervision, start, and follow-up measurements), length of the intervention, type and characteristics of the control intervention used (frequency and intensity), primary and secondary outcome measures, main results, and authors’ conclusions about the effectiveness. Types of exercise intervention were divided into three categories as done by Wijnen et al. [14]: strengthening (explicitly aimed at improving muscle strength), endurance, and functional exercise (focussed on training functional tasks but not explicitly on improving muscle strength or endurance).

#### Therapeutic validity

Therapeutic validity of included exercise interventions was assessed using the ‘CONTENT scale for therapeutic validity’ of Hoogeboom et al. [21]. The CONTENT scale is used to assess therapeutic validity of exercise programmes related to five main domains: Patient eligibility, Competences and setting, Rationale, Content, and Adherence. These domains add up to nine items, divided into 17 sub-items. Every item was dichotomously rated (yes or no). An item was only scored ‘yes’ if all sub-items for that topic were scored ‘yes’, following the developers’ recommendations [21]. Each item that scored ‘yes’ was awarded one point. Scores on all nine items were summed, resulting in a total score ranging from 0 to 9. Studies that received a total score ≥ 6 were judged to be therapeutically valid [21].

### Data analysis

Two blinded authors (HWvK & GHS) read the included full-text articles and extracted relevant data independently using Covidence. In studies that included patients outside the current review’s target group, only the data relevant to the current review were extracted. Extracted data from both authors were compared and differences were resolved during a consensus meeting. A third reviewer (IvdAS) was consulted to give a final judgement if disagreement persisted. The same methodology was applied for assessing therapeutic validity and methodological quality.

Heterogeneity of outcome measures and reported measurement units precluded meta-analysis. Hence a formal narrative data synthesis was performed to evaluate the included studies’ results and to give recommendations for future research. Individual studies’ outcomes were divided into categories and presented accordingly as ‘Joint and muscle function’ (e.g., range of motion (ROM), muscle strength), ‘Functional performance’ (e.g., musculoskeletal tumour society (MSTS) score, Toronto extremity salvage score (TESS)), and ‘Other’ (e.g., compliance, pain). Interventions’ effectiveness was determined only for studies that included a control group, and only for the most-reported measure per outcome category. Per study, mean differences (MD) were calculated between the last-reported measurements of the intervention and control group.

According to the Synthesis Without Meta-analysis (SWiM) reporting guidelines, it is recommended to assess the certainty of narrative synthesis findings [29]. To this end, the Grading of Recommendations, Assessment, Development, and Evaluations (GRADE) framework was applied [30]. The studies were grouped by exercise intervention type (strengthening, endurance, and functional exercise). Per intervention type, the certainty of evidence of the most-reported measure per outcome category was assessed [30].

## Results

### Study selection

**Fig. 1:**
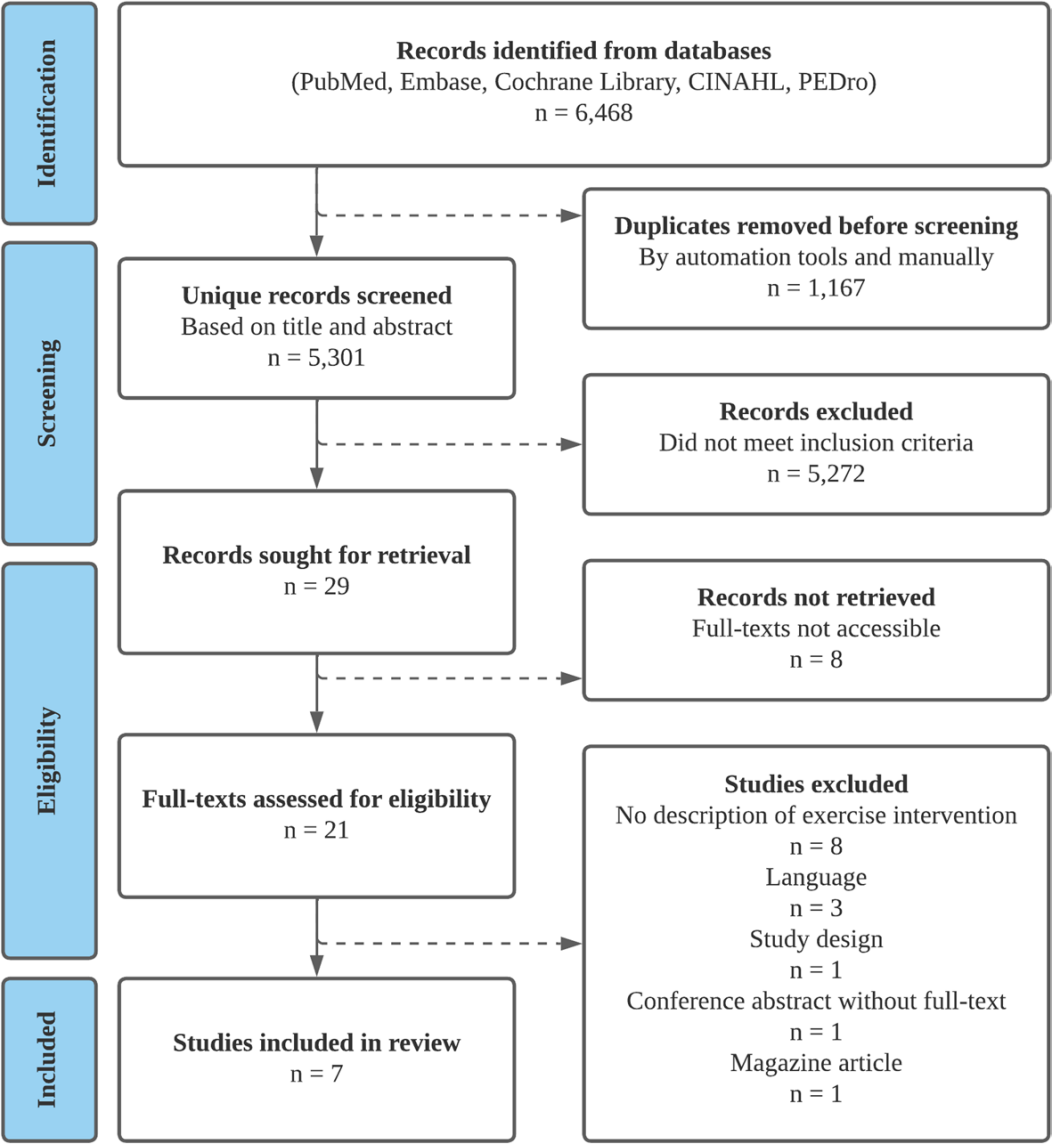
PRISMA flowchart of the inclusion process

Of the 6,468 records identified, 1,167 were duplicates. Out of 5,301 unique records, 5,272 were excluded based on title and abstract, with an agreement of 99.5% between reviewers. Twenty-nine remained as being potentially relevant articles and were thus screened based on full-text. Despite every effort made to contact authors and libraries, eight articles could not be retrieved. Out of 21 full-texts, seven articles were eligible and thus included in the current systematic review (Fig. 1). References excluded during full-text screening accompanied by reason for exclusion are listed in Additional file 2. Manual search of included articles’ reference lists revealed no additional eligible articles.

### Methodological quality

Absolute agreement between the two reviewers was achieved in 148 out of 189 items (78.3%) of the Downs & Black checklist on methodological quality (Table 2). Median quality score was 18 (range 14–21) out of 28. Seventeen items could not be determined due to insufficient reporting. Three studies were considered of good quality, three fair, and one poor.

**Table 2:** Methodological quality assessment (Downs & Black checklist)

Study	Reporting										External validity			Internal validity – Bias and Confounding													Power	Total score (n, %)^c^
	1	2	3	4	5^a^	6	7	8	9	10	11	12	13	14	15	16	17	18	19	20	21	22	23	24	25	26	27^b^	
Morri [31]	Y	Y	Y	Y	Y	Y	Y	Y	N	Y	Y	Y	Y	N	N	Y	U	Y	Y	Y	Y	Y	N	N	N	Y	N	20 (71)
Morri [32]	Y	Y	Y	Y	Y	Y	Y	Y	N	Y	Y	Y	Y	U	N	Y	Y	Y	Y	Y	Y	Y	N	N	U	Y	N	21 (75)
Morri [33]	Y	Y	Y	Y	Y	Y	Y	Y	N	Y	Y	Y	Y	N	U	Y	Y	Y	Y	Y	Y	N	N	N	Y	Y	N	21 (75)
Pitera [34]	Y	Y	Y	Y	P	Y	Y	Y	N	N	U	U	Y	N	U	Y	U	Y	Y	Y	Y	Y	N	N	U	Y	N	16 (57)
Shehadeh [35]	Y	Y	N	Y	Y	Y	Y	N	N	N	Y	N	Y	N	U	Y	N	Y	U	Y	Y	Y	N	N	N	U	N	14 (50)
Tsauo [36]	Y	Y	Y	N	Y	Y	Y	N	Y	N	Y	Y	Y	N	N	Y	N	Y	Y	Y	N	Y	N	N	Y	Y	N	18 (64)
Zhang [37]	Y	Y	Y	Y	N	Y	Y	N	Y	N	U	N	Y	U	U	Y	Y	Y	Y	Y	Y	Y	Y	U	U	Y	N	17 (61)
‘Yes’ (%)	100	100	86	86	79	100	100	57	29	43	71	57	100	0	0	100	43	100	86	100	86	86	14	0	29	86	0	

Yes (Y) = 1 point; No (N) = 0 points; Unable to determine (U) = 0 points

^a^Yes = 2 points; Partially (P) = 1 point; No = 0 points

^b^Adjusted question (Power analysis reported?): Yes = 1 point; No = 0 points

^c^Scale for quality scores: ≤ 14 = Poor; 15–19 = Fair; 20–25 = Good; 26–28 = Excellent

### Study characteristics

Included studies were published between January 2006 and April 2019 (Table 3). Five studies had a cross-sectional design [31, 32, 34–36], the remaining two articles were a clinical [33] and randomised controlled trial [37]. Three articles included a control group [33, 36, 37]. Sample sizes ranged between 22 to 59 in the intervention groups and 15 to 30 in the control groups. Of the articles reporting tumour morphology, four included primary lower limb bone tumours only [31–33, 37], while two included patients with either primary or metastatic cancer [34, 35]. Out of 214 cases specifying tumour location, the most frequent location was distal femur (59%), followed by proximal tibia (24%) and proximal femur (10%). Location was not reported in 47 cases. Only one study reported cancer grade [37]. Modular knee endoprostheses were utilised in five studies [31–33, 36, 37], modular proximal femur prostheses in one [34], and in one study the endoprostheses types or bone grafts used were not described [35]. Complications were reported in five studies [31–34] and consisted mainly of infections, mechanical failures, chemotherapy side effects, and knee stiffness. No skeletal-related events caused by the interventions were reported.

**Table 3:** Summary of the included studies (*n* = 7)

Study, Year Country Design	N	Sex (% F)	Age (y)	BMI (kg/m^2^)	Tumour description (n)^a^	Surgery type (n)^b^; Resection amplitude (cm)	Complications (% of N)	Outcome measures^c^		
								Joint and muscle function	Functional performance	Other
Morri 2018 [31] Italy Cross-sectional	IG: 30	33	19 (9–60)	NR	Primary OS (25) or ES (55) in DF (19) and PT (11)	MKP (30); 14.5 (11–30)	30	• Knee ROM^e^ • Quadriceps strength (MRC scale)^e^	• TESS^e^ • TUG^f^ • 6mWT^e^	
Morri 2018 [32] Italy Cross-sectional	IG: 27	33	19 (9–60)	22 (14–34)	Primary OS/SCS (22) or ES (5) in DF (19) or PT (8)	MKP (27); NR	30		• MSTS^e^	• Compliance^e^ • NRS satisfaction^e^
Morri 2019 [33] Italy CCT	IG: 22 CG: 15	IG: 23 CG: 33	IG: 21 (30) CG: 21 (15)	NR	Primary OS (31) or CS/ES (6) in DF (27) or PT (10)	MKP (37); IG: 15 (9) CG: 15 (7)	IG: 14 CG: 27	• Knee ROM^e^ • Quadriceps strength (MRC scale)^e^	• MSTS^e^ • TESS^e^ • TUG^f^ • 6mWT^e^ • 10mWT^e^	• CoM speed^f^
Pitera 2017 [34] Poland Cross-sectional	IG: 42	55	63 ± 11	NR	Primary or metastatic GCT (1), LS (1), or NR (40) in PF (13) or NR (29)	C-MPFP (36), UC-MPFP (8); NR (8–26)	14		• MSTS^e^ • HHS^e^ • Custom system^e^	• VAS-pain^f^
Shehadeh 2013 [35] Jordan Cross-sectional	IG: 59	46	24 (5–60)	NR	Primary or metastatic OS (28), ES (13), CS (5), or other (13) in PF (6), DF (21), PF and DF (2), TF (2), or other (14)	Endoprosthesis (49), bone graft (5), or no replacement (5); NR	12		• MSTS-ISOLS^e^	
Tsauo 2006 [36] Taiwan Cross-sectional	IG: 20 CG: 20^d^	NR	IG: 22 ± 7 CG: 22 ± 7	NR	IG: In DF (13) or PT (7) CG: NA	MKP (20); DF: 16 ± 4 PT: 16 ± 2	NR	• Knee ROM^e^ • Isokinetic knee muscle strength^e^	• MSTS^e^ • Gait evaluation	
Zhang 2016 [37] China RCT	IG: 30 CG: 30	IG: 40 CG: 27	IG: 25 ± 6 CG: 24 ± 8	NR	Primary OS (42), GCT (14) or ES (2), in DF (26), PT (16), or NR (18), Enneking grade I or II	MKP (60); NR	NR	• Knee ROM^e^	• First time off bed^f^ • HSS^e^	• Compliance^e^ • Grade-A wound recovery^e^

‘Age’ and ‘Resection amplitude’ are shown as median (range or IQR), or mean ± SD

*CG* control group, *IG* intervention group, *NA* not applicable, *NR* not reported, *CCT* clinical controlled trial, *RCT* randomised controlled trial

^a^OS, osteosarcoma; ES, Ewing sarcoma; SCS, spindle cell sarcoma; CS, chondrosarcoma; GCT, giant cell tumour; LS, leiomyosarcoma; DF, distal femur; PF, proximal femur; PT, proximal tibia; TF, total femur

^b^MKP, modular knee prosthesis; C-MPFP, cemented modular proximal femur prosthesis; UC-MPFP, uncemented modular proximal femur prosthesis

^c^ROM, range of motion; MRC, medical research council; MSTS, musculoskeletal tumour society; TESS, Toronto extremity salvage score; TUG, timed up-and-go; 6mWT, 6-min walk test; 10mWT, 10-m walk test; HHS, Harris hip score; ISOLS, International Society of Limb Salvage; HSS, hospital for special surgery knee score; NRS, numeric rating scale; CoM, centre of mass; VAS, visual analogue scale

^d^Control group consisted of healthy controls

^e^Higher scores indicate better performance

^f^Lower scores indicate better performance

All included studies investigated the effects of functional exercise that started on the first postoperative day. Intervention length varied between two weeks [37] and eight months [35]. Follow-up periods ranged from six weeks [34] to five years [36]. All articles reported functional performance outcome measures, of which MSTS was reported most frequently [32–36], followed by TESS [31, 33], timed up-and-go (TUG) [31, 33], 6-min walk test (6mWT) [31, 33] 10-m walk test (10mWT) [33], Harris hip score (HHS) [34], a custom-made functional performance scoring system [34], gait evaluation (including walking speed) [36], the hospital for special surgery (HSS) knee score [37], and, last, the number of patients that had been out of their beds for the first time on the third, seventh, and fourteenth postoperative day [37]. Outcome measures in the category ‘Joint and muscle function’ were reported in four out of seven articles (57%), with knee ROM being reported most frequently [31, 33, 36, 37], followed by quadriceps strength on the medical research council (MRC) scale [31, 33] and isokinetic muscle strength [36]. ‘Other’ outcome measures were utilised in four studies (57%) and included intervention compliance [32, 37], satisfaction [32], pain [34], balance [33], and number of patients with grade-A wound recovery [37].

### Characteristics of performed interventions

Aims of the articles were similar, as all seven investigated the effects of functional exercise on postoperative outcomes. The overarching goal was to minimise surgery-related disabling effects and to achieve the best possible recovery of residual abilities [31–37]. However, the interventions conducted to achieve this goal varied between studies, as outlined below and in Table 4.

**Table 4:** Characteristics of the exercise interventions

Study	Exercise intervention						Control intervention		
	Setting	Exercise type	Session duration	Frequency	Programme start and duration	Intensity	Description	Frequency	Intensity
Morri [31]	Inpatient	Functional	45	2/d, 2-6d/w, every 3w	1dPO; 6mo	Individualised ≤ 2wPO: PWB > 2wPO: FWB	NA	NA	NA
Morri [32]	Supervised, inpatient	Functional	45	2/d, 2-6d/w, every 3w	1dPO; 6mo	Individualised PWB or FWB	NA	NA	NA
Morri [33]	Inpatient	Functional	45	2/d, 2-6d/w, every 3w	1dPO; 6mo	NR	Retrospectively chosen patients, usual care with functional exercise	2/d, 2-6d/w, every 3w	NR
Pitera [34]	Inpatient	Functional	Protocol Shehadeh [35]	Protocol Shehadeh [35]	1dPO; NR	≤ 6wPO: PWB > 6wPO: FWB	NA	NA	NA
Shehadeh [35]	Supervised, inpatient and outpatient	Functional	NR	< 6wPO: 2–4/w > 6wPO: 1–2/w	1dPO; 4-8mo	NR	NA	NA	NA
Tsauo [36]	Inpatient	Functional	NR	NR	< 1dPO; > 1y	NR	Healthy controls and patients’ sound knees, no intervention	NA	NA
Zhang [37]	Inpatient	Functional	15–45	4 or 8/d	6hPO; > 2w	Prehab: 10 reps, 5–10 sets ≥ 2wPO: 20 reps	Prospectively chosen patients, CPM	2–3 h/d (for 2w)	≥ 30°, 45 s cycle

*NA* not applicable, *NR* not reported, *h* hour(s), *d* day(s), *w* week(s), *mo* month, *y* year, *PO* postoperative, *PWB* partial weight bearing, *FWB* full weight bearing, *CPM* continuous passive motion

Intervention settings were either inpatient [31–34, 36, 37] or a combination of inpatient and outpatient [35]. One study also encouraged participants to exercise at home [36]. Authors of two articles reported that the intervention was supervised by a healthcare professional [32, 35], while in the remaining articles supervision was not clearly addressed [31, 33, 34, 36, 37]. All studies used ‘physical therapy’ as their training modality. Types of exercises reported in the studies were knee ROM exercises and quadriceps-strengthening exercises [31–37]; hamstring-strengthening exercises [35, 37]; hip ROM training [34, 36, 37]; ankle exercises [34, 35, 37]; toe-touch weight bearing [35]; proprioceptive exercises [31]; closed-eye training or dual-task exercises [31, 33]; load-shifting with or without use of a Wii Balance Board [31, 33]; various other exercises for balance training [33]; ambulation training [32, 34]; transfer and crutch-walking training [36, 37]; and continuous passive motion (CPM) [37]. One study did not provide examples of the exercises performed [32]. In three studies, more progressive physical therapy was taught after removal of an immobiliser or functional brace [34–36].

Reported exercise frequencies ranged from a maximum of eight times daily [37] to a minimum of one session per week [35]. Two studies did not report on session frequency [34, 36]. Exercise intensity was either not clearly described or not reported [33, 35, 36]. Progressive partial weight bearing to eventually full weight bearing (either six weeks [34] or two months [31] postoperatively, or individualised [32]) was reported most frequently. One study reported sets of repetitions, with 5–10 sets of 10 repetitions preoperatively to 20 repetitions postoperatively [37]. Session duration varied between 15 [37] and 45 min [31–33, 37], and was not specified in three studies [34–36].

In one of the three studies that included a control group, the group was retrospectively chosen from the hospital’s database and comparable to the intervention group patients [33]. This control group also performed ROM and quadriceps-strengthening exercises, but no balance training [33]. In another study the control group consisted of similar patients and was prospectively randomly chosen [37]. These patients performed CPM only [37]. In the last study that included a control group, the group consisted of sex- and age-matched healthy controls who did not follow an intervention [36].

#### Therapeutic validity

In 59 out of 63 items (93.7%) assessed for the CONTENT scale, absolute agreement between both raters was achieved (Table 5). Disagreements were solved during consensus discussion without consulting the third assessor. The median therapeutic validity score was 5 (range 1–5) out of 9. None of the seven interventions could be labelled as being therapeutically valid according to the ≥ 6 cut-off score [21].

**Table 5:** Therapeutic validity assessment (CONTENT scale)

Study	Patient eligibility		Setting and therapist	Rationale		Content			Adherence	Total score (n, %)
	Described	Adequate		Study	Intervention	Intensity	Monitored	Personalised		
Morri [31]	N	Y	N	Y	Y	Y	Y	N	N	5 (56)
Morri [32]	N	Y	N	Y	N	Y	Y	N	Y	5 (56)
Morri [33]	N	Y	N	Y	Y	Y	Y	N	N	5 (56)
Pitera [34]	Y	Y	N	N	N	N	N	N	N	2 (22)
Shehadeh [35]	N	Y	N	Y	Y	N	Y	N	N	4 (44)
Tsauo [36]	N	Y	N	N	N	N	N	N	N	1 (11)
Zhang [37]	Y	Y	N	N	N	Y	Y	N	Y	5 (56)
‘Yes’ (%)	29	100	0	57	43	57	71	0	29	

Total score ≥ 6 indicates high therapeutic validity

### Effectiveness of interventions

In the studies that included a control group of patients, knee ROM results were in favour of the intervention groups (MD = 10–15°; Table 6). Morri et al.’s [33] control group had a higher MSTS score than the intervention group (MD = -5%). Zhang et al.’s [37] subject compliance with the exercise program was higher in the intervention group than the control group (MD = 30%).

**Table 6:** Effectiveness per outcome measure for studies that included a control group, and certainty of evidence (GRADE)

Study^a^	Groups		Difference between groups	GRADE
	Intervention	Control	Intervention – Control	
Joint and muscle function (Knee ROM, °)				Very low
Morri [33]	110	100	10 (+ 10%)	
Zhang [37]	95	80	15 (+ 19%)	
Functional performance (MSTS, %)				Very low
Morri [33]	78	83	-5 (-6%)	
Other (Compliance, %)				Very low
Zhang [37]	83	53	30 (+ 57%)	

^a^Length of follow-up: Morri [33], 12 months; Zhang [37], 6 months

Twelve months after the balance training, Morri et al.’s [33] intervention group scored significantly better on the 10mWT (median (IQR): 1.48 (0.5) m/s) than the control group (median (IQR): 1.26 (0.6) m/s; *P* = 0.022). The intervention group’s centre of mass speed was significantly slower (median (IQR): 4.8 (2.5) mm/s) than the control group’s (median (IQR): 9.3 (5.2) mm/s; *P* = 0.005). No other significant differences were found between groups twelve months postoperatively.

In the pre- and postoperative physical therapy intervention, Zhang et al. [37] found that the mean knee flexion ROM in the intervention group was significantly greater than the control group at two weeks and three and six months postoperatively (81.2 ± 1.8° vs. 59.3 ± 6.6°, *P* < 0.01; 90.7 ± 7.6° vs. 70.3 ± 6.5°, *P* < 0.05; 95.4 ± 6.2° vs. 71.0 ± 4.3°, *P* < 0.05, respectively). The intervention group’s HSS knee score was significantly higher at each follow-up point (72.4 ± 7.7 vs. 34.6 ± 6.5, *P* < 0.01; 80.6 ± 6.6 vs. 50.4 ± 6.1, *P* < 0.01; 87.7 ± 8.3 vs. 71.0 ± 4.3, *P* < 0.05, respectively). Complete compliance was significantly higher in the intervention group (*n* = 25, 83.3%) than in the control group (*n* = 16, 53.3%; *P* < 0.05). Last, the number of patients with grade-A wound healing was significantly higher in the intervention group (*n* = 22, 73.3%) than the control group (*n* = 17, 56.7%; *P* < 0.05) seven, but not fourteen days postoperatively (*n* = 27, 90% vs. *n* = 26, 86.7%; *P* > 0.05).

#### GRADE

All studies conducted functional exercise interventions. These studies’ quality of evidence was *very low* for knee ROM, MSTS, and compliance. As the majority of studies was non-randomised, the starting point of the evidence was *low* quality for each outcome according to the GRADE framework [30]. Lack of blinding, inconsistent length of follow-up, varying exercises between studies, and small sample sizes increased the risk of bias, inconsistency, and imprecision. Quality was therefore downgraded for every outcome. Since downgrading *very low* is impossible and upgrading was not applicable, the eventual quality of evidence for all three outcomes was *very low* (Table 6).

## Discussion

This systematic review assessed the therapeutic validity of exercise interventions after lower LSS for bone cancer from the literature, and the exercise interventions’ effectiveness. It was found that therapeutic validity of exercise interventions in this specific population was insufficient. Also, the certainty of evidence was considered very low even though all but one study were considered of at least adequate methodological quality. No clear evidence was found for the effectiveness of the included exercise interventions after lower LSS for bone cancer due to heterogeneity in characteristics of exercise and control interventions, lengths of follow-up, and study methods, including outcome measures.

### Therapeutic validity

Our finding that exercise interventions after lower LSS have insufficient therapeutic validity is comparable with interventions in related patient populations. For interventions following primary joint replacement, Wijnen et al. [14] found one out of twenty interventions to be therapeutically valid, whereas Hoogeboom et al. [21] found none in twelve. In the current review, one reason for the low validity scores was that none of the assessed interventions satisfied the item about personalisation and contextualisation of the exercise to the individual participants. The item is fulfilled if the goals of exercising not only match the patient’s bodily functions and structures, activities, and participation levels but also their personal and environmental factors [21]. These requirements are based on the ICF model, which underlines the importance of environmental factors such as personality traits and the presence or absence of social support, given their major role in patients’ well-being and recovery [18, 38]. Also, just one of the interventions reported on the therapist and setting in which therapy is provided, while it is known that both therapist selection and therapeutic setting influence treatment effects [39]. As there is great variation in patients’ individual functional levels and contextual factors, individualised exercise yields better outcomes than generalised exercise training and should be strived for [40]. This may be especially true in a vulnerable and heterogeneous patient group such as bone cancer survivors after lower LSS.

The inadequate therapeutic validity of exercise interventions hardens conclusive interpretation of their effectiveness. Actual effects could be overestimated in studies with a high risk of bias because of factors like improper patient or modality selection. Conversely, actual effects could be underestimated when the patient sample was functioning relatively well during baseline measurements, which gave them less room to improve over time. Two meta-analyses combining the results of studies in other patient populations confirm this problem, as no significant association was found between therapeutic validity scores and interventions’ effectiveness [21, 41].

The difficulty of validating therapeutic interventions may arise from the CONTENT scale scores not being representative, as it is uncertain whether a low score means that the scale’s requirements were truly not met or whether required information was insufficiently reported [21]. Therefore, to correctly interpret exercise interventions’ effectiveness it is necessary for authors to properly report on all items of the CONTENT scale. It has been recommended that exercise interventions be described in sufficient detail to enable readers to understand how the intervention was conducted exactly [20]. Reporting with insufficient detail prevents reviews like ours from drawing well-founded conclusions. As physical therapists and other clinicians base their own interventions on findings like ours, it is important to substantiate which exercises are prescribed at what intensity and why, rather than simply mention that physical therapy measures were administered. However, the CONTENT scale is a relatively new measure, which may imply that authors of the included articles were not yet aware of the required level of detail to sufficiently describe interventions. For the score to be more representative of interventions’ actual therapeutic validity, it is highly recommended that future studies use the CONTENT scale as a template for planning and reporting exercise interventions. Correct reporting allows clinicians to translate knowledge gained through research into daily practice, which in the end is the goal of this type of research.

### Effectiveness of interventions

In multiple patient populations, exercise training has been shown to have positive effects on patients’ functioning and overall well-being [15]. No such conclusion can be currently drawn for patients after lower LSS as treatment for bone cancer. The heterogeneity in intervention characteristics and study designs, such as the presence or absence of a control group and its features, and different outcome measures between studies resulted in very low quality of evidence, which hampers determining the effectiveness of the interventions presented. This finding concords with previous research into the role of exercise in cancer treatment, which concluded that the majority of intervention studies are lacking sufficient quality in reporting and transparency of exercise prescription and guidelines [42]. Studies in the current review all administered functional exercise, which was not part of our inclusion criteria, confirming the need to improve functional impairments in the investigated patient population [12]. This lack of variety prevented us from comparing the effectiveness of different exercise types. As a result, the GRADE certainty of evidence assessment could only be performed on outcomes after functional exercise. A few studies included a control group or even pre-surgery measurements, making it impossible to establish the intervention’s relative effect. Therefore, our hypothesis on the matter can be neither confirmed nor rejected at this point. Ultimately, better-quality studies with uniformity in methodological approach, including a control group are warrented to sufficiently conclude on any exercise interventions’ effectiveness for patients after lower LSS for bone cancer.

The oldest study included was published in 2006, which indicates that exercise intervention research in this particular patient population has not been a consideration for that long. Given prescribers’ apprehensions about exercise training inducing skeletal-related events [17], it is worth noting that no adverse events of this type are reported so concerns seem unwarranted. It is recommended that clinicians include this notion in their thinking, and once the exercise interventions’ effectiveness is more certain, update current rehabilitation policies by including exercise.

### Methodological quality

While six out of seven studies were of at least fair methodological quality, future research should focus on improving methodological quality to reduce risk of bias. On some items the included studies scored poorly – albeit sometimes due to the nature of their design. Blinding participants in intervention studies is not always possible. This notwithstanding, future studies should strive to blind participants, if applicable within the study’s design, or at least blind those measuring the outcomes to improve the quality of their results.

Another shortcoming of the included intervention studies was the lack of a priori power analyses performed to calculate required sample sizes. It can be argued that the bone cancer population is rather small, never mind the population of patients with bone cancer that requires lower extremity LSS. It remains difficult to include the required number of patients for sufficient power in a single-centre study. We therefore recommend conducting multi-centre studies on a national, European, or even global scale. Upscaling will facilitate larger sample sizes, greater generalisability, and thus external validity, plus result in greater evidence certainty and study quality.

## Strengths and limitations

To our knowledge, this systematic review is the first to assess therapeutic validity of exercise interventions after lower LSS as treatment for bone cancer. The CONTENT scale [21] results provide insight into interventions’ quality, or at least their quality of reporting. In addition, this review included critical appraisal of the methodological quality of the included studies. Despite meta-analysis being impossible, the reader was provided with tools for how to interpret the results by applying GRADE [30], following the most recent SWiM guidelines as per current recommendations [29]. Another strength was that the review’s protocol, which followed current PRISMA [24] recommendations, had been published [23]. Additionally, multiple relevant databases were searched, and the search strategy for each of these was refined by an experienced scientific librarian.

Limitations of this study include that despite a profound search, relevant articles might have been missed due to language shortcomings or due to the search strategies’ structure. However, a second scientific librarian checked and revised one random search strategy and no additional relevant articles were identified. Second, the assessments of therapeutic validity and study quality throughout the CONTENT scale and Downs & Black checklist, respectively, might have assessed the quality of reporting over that of the intervention or study actually conducted. Still, more well-designed RCTs examining the effects of different types of exercise interventions on physical activity, physical activity behaviour and/or functioning are needed to provide more, homogeneous evidence. This evidence will eventually help both researchers and clinicians, such as physical therapists, to understand and exploit the use of exercise after lower LSS in patients with bone cancer.

## Conclusions

With the combination of deficient exercise interventions’ therapeutic validity and very low certainty of evidence, no distinct conclusions can be drawn about the effect of exercise interventions on physical activity, physical activity behaviour and/or functioning in patients undergoing surgical management of lower extremity bone cancer. However, the included studies show promising results, so the use of exercise interventions in this population is worth further investigating. The small sample sizes, methodological shortcomings, and possible insufficient reporting in available articles indicate that larger-scale, prospective, long-term follow-up studies in patients with lower limb bone tumours, using a standardised core outcome set including not only functional outcomes but participation and QoL too, are needed. The CONTENT scale should be used as a template to avert insufficient reporting.

## Clinical messages

- Lower limb-salvage surgery for bone cancer leaves patients with restricted physical functioning and quality of life.
- Though evidence is limited, functional exercise may contribute to improving joint and muscle function in this patient group.
- Exercise does not seem to exacerbate skeletal-related events after lower limb-salvage surgery.

## Supplementary Information

**Additional file 1.** Search Strategies.

**Additional file 2.** Excluded Full-texts.

**Additional file 3.** PRISMA Checklist.

## Data Availability

The dataset supporting the conclusions of this article is included within the article and its additional files.

## Abbreviations

10mWT: 10-Metre walk test
6mWT: 6-Minute walk test
ADL: Activities of daily living
CONTENT: Consensus on therapeutic exercise training
CPM: Continuous passive motion
GRADE: Grading of recommendations, assessment, development and evaluations
HHS: Harris hip score
HSS: Hospital for special surgery
ICF: International classification of functioning, disability and health
LSS: Limb salvage surgery
MD: Mean difference
MRC: Medical research council
MSTS: Musculoskeletal tumour society
PRISMA: Preferred reporting items for systematic reviews and meta-analyses
ROM: Range of motion
SWiM: Synthesis without meta-analysis
TESS: Toronto extremity salvage score
TUG: Timed up-and-go
