# Supplementary material for: Therapeutic validity and effectiveness of exercise interventions after lower limb-salvage surgery for sarcoma: a systematic review
Source: BMC Musculoskelet Disord. 2023 Mar 23;24:216. doi: 10.1186/s12891-023-06315-y (PMC10035240; doi:10.1186/s12891-023-06315-y)
Supplement: Supplementary file 1 — Additional file 1. Search Strategies. [file 12891_2023_6315_MOESM1_ESM.pdf]

PubMed

("Bone Neoplasms"[Mesh] OR bone neoplasm*[tiab] OR bone cancer*[tiab] OR bone sarcoma*[tiab] OR bone carcinoma*[tiab] OR bone tumo*[tiab] OR "Osteosarcoma"[Mesh] OR osteosarcoma*[tiab] OR ewing sarcoma*[tiab] OR chondrosarcoma*[tiab] OR malignant fibrous histiocytoma*[tiab] OR fibrosarcoma*[tiab] OR chordoma*[tiab] OR giant cell tumor of the bone[tiab] OR gctb[tiab] OR femur metastas*[tiab])

AND

("Surgical Procedures, Operative"[Mesh] OR "surgery" [Subheading] OR surger*[tiab] OR operat*[tiab] OR arthroplast*[tiab] OR orthoped*[tiab] OR joint replac*[tiab] OR resection*[tiab] OR reconstruct*[tiab] OR allograft*[tiab] OR APC[tiab])

AND

("Rehabilitation"[Mesh] OR "rehabilitation" [Subheading] OR "Physical Therapy Modalities"[Mesh] OR physical therap*[tiab] OR physiotherap*[tiab] OR "Exercise"[Mesh] OR exercis*[tiab] OR training*[tiab] OR rehabil*[tiab])

EMBASE

('bone tumor'/exp OR 'osteosarcoma'/exp OR (‘bone neoplasm*’ OR ‘bone cancer*’ OR ‘bone sarcoma*’ OR ‘bone carcinoma*’ OR ‘bone tumo*’ OR osteosarcoma* OR ‘ewing sarcoma*’ OR chondrosarcoma* OR ‘malignant fibrous histiocytoma*’ OR fibrosarcoma* OR chordoma* OR ‘giant cell tumor of the bone’ OR gctb OR ‘femur metastas*’):ab,ti)

AND

('surgery'/exp OR (surger* OR operat* OR arthroplast* OR orthoped* OR ‘joint replac*’ OR resection* OR reconstruct* OR allograft* OR APC):ab,ti)

AND

('rehabilitation'/exp OR 'physiotherapy'/exp OR 'exercise'/exp OR (‘physical therap*’ OR physiotherap* OR exercis* OR training* OR rehabil*):ab,ti)

Cochrane Library

(“bone neoplasm*” OR “bone cancer*” OR “bone sarcoma*” OR “bone carcinoma*” OR “bone tumo*” OR osteosarcoma* OR “ewing sarcoma*” OR chondrosarcoma* OR “malignant fibrous histiocytoma*” OR fibrosarcoma* OR chordoma* OR “giant cell tumor of the bone” OR gctb OR “femur metastas*”)

AND

(surger* OR operat* OR arthroplast* OR orthoped* OR “joint replac*” OR resection* OR reconstruct* OR allograft* OR APC)

AND

(rehabil* OR “physical therap*” OR physiotherap* OR exercis* OR training*)

CINAHL

((MH "Bone Neoplasms+") OR TI (“bone neoplasm*” OR “bone cancer*” OR “bone sarcoma*” OR “bone carcinoma*” OR “bone tumo*” OR osteosarcoma* OR “ewing sarcoma*” OR “chondrosarcoma*” OR “malignant fibrous histiocytoma*” OR fibrosarcoma* OR chordoma* OR “giant cell tumor of the bone” OR gctb OR “femur metastas*” OR “spinal metastas*”) OR AB (“bone neoplasm*” OR “bone cancer*” OR “bone sarcoma*” OR “bone carcinoma*” OR “bone tumo*” OR osteosarcoma* OR “ewing sarcoma*” OR “chondrosarcoma*” OR “malignant fibrous histiocytoma*” OR fibrosarcoma* OR chordoma* OR “giant cell tumor of the bone” OR gctb OR “femur metastas*”))

AND

((MH "Surgery, Operative+") OR TI (surger* OR operat* OR arthroplast* OR orthoped* OR “joint replac*” OR resection* OR reconstruct* OR allograft* OR APC) OR AB (surger* OR operat* OR arthroplast* OR orthoped* OR “joint replac*” OR resection* OR reconstruct* OR allograft* OR APC))

AND

((MH "Rehabilitation+") OR (MH "Physical Therapy+") OR (MH "Exercise+") OR TI (“physical therap*” OR physiotherap* OR exercis* OR training* OR rehabil*) OR AB (“physical therap*” OR physiotherap* OR exercis* OR training* OR rehabil*))

PEDro

Abstract & Title:	Exercise intervention / Intervention / Limb-salvaging surgery
Body Part:	Lower leg or knee / Thigh or hip
Subdiscipline:	Oncology / Orthopaedics
Method:	Clinical Trial
