# Supplementary material for: Therapeutic validity and effectiveness of exercise interventions after lower limb-salvage surgery for sarcoma: a systematic review
Source: BMC Musculoskelet Disord. 2023 Mar 23;24:216. doi: 10.1186/s12891-023-06315-y (PMC10035240; doi:10.1186/s12891-023-06315-y)
Supplement: Supplementary file 2 — Additional file 2. Excluded Full-texts. [file 12891_2023_6315_MOESM2_ESM.pdf]

Unretrievable:

- Guo, Z. et al. Follow-up evaluation of the artificial joint reconstructed functional rehabilitation after knee joint peripheral tumor resection. Chinese Journal of Clinical Rehabilitation, 2006; 10(4): 42-43.
- Najenson, T., Levy, M. Rehabilitation for amputees following malignant tumours. Scandinavian journal of rehabilitation medicine, 1970; 2(1): 7-12.
- Protsenko, V.V. & Tolstopiatov, B.A. Patient medical rehabilitation osteoplastic operations using hydroxylapatite-based porous ceramic. Likars'ka sprava / Ministerstvo okhorony zdorov'ia Ukraïny, 1999; (5): 71-73.
- Sacco, F. et al. Function of lower extremity soft tissue sarcomas. Minerva Ortopedica e Traumatologica, 2006; 57(3): 157-163.
- Shi, Z.-J. et al. Limb salvage and limb function reconstruction in II B and III stage osteosarcoma patients. Chinese Journal of Clinical Rehabilitation, 2003; 7(23): 3232-3233.
- Sipus, N. Rehabilitation of patients with malignant tumors of the extremities (Serbocroatian). Libri Oncologi, 1974; 3(3): 225-228.
- Ward, T.A. Physiotherapy management of lower limb bone and joint massive replacements for the treatment of bone tumour. Physiotherapy, 1986; 72(9): 440-442.
- Zhao, T.-B. et al. Significance of CPM for knee joint rehabilitation after treatment of bone tumors near knee with microwave-induced hyperthermia. Chinese Journal of Clinical Rehabilitation, 2002; 6(22): 3392-3393.

No description of exercise intervention:

- Morri, M. et al. Which factors are associated with the functional recovery in patients undergoing endoprosthetic knee reconstruction following bone tumour resection? - A observational study. Arch Physiother, 2018; 8(): 11.
- Ogilvie, C.M. et al. Long-term results for limb-salvage with osteoarticular allograft reconstruction. Clinical Orthopaedics and Related Research, 2009; 467(10): 2685-2690.
- Ogilvie, C.M. et al. Functional outcome of endoprosthetic proximal femoral replacement. Clinical Orthopaedics and Related Research, 2004; (426): 44-48.
- Penna, V. et al. A new approach to partial knee endoprosthesis in primary bone sarcomas. Rev Bras Ortop, 2009; 44(1): 46-51.
- Pitera, T. et al. Assessment of Physical Performance and Early Treatment Outcomes after Implantation of Modular Prostheses of Femoral and Tibial Shaft. Ortopedia, traumatologia, rehabilitacja, 2019; 21(5): 349-358.
- Prajapati, A. et al. Is minimal reconstruction (meshplasty) adequate to restore ankle function after excision of distal fibula tumors? Journal of Clinical Orthopaedics and Trauma, 2020; 11(3): 467-470.
- Tan, P.K., Tan, M.H. Functional outcome study of mega-endoprosthetic reconstruction in limbs with bone tumour surgery. Ann Acad Med Singap, 2009; 38(3): 192-196.
- Thorpe, W. et al. A prospective study of the rehabilitation of the above-knee amputee with rigid dressing. Comparison of immediate and delayed ambulation and the role of physical therapists and prosthetists. Clin Orthop Relat Res, 1979; (143): 133-137.

Language:

- Dubousset, J.F. et al. Retraining the knee after osteosarcoma with resection and prosthesis replacement. Cahiers de Kinesitherapie, 1984; NO. 107:41-57
- Grushina, T.I., Teplyakov, V.V. Physiotherapy in early rehabilitation with bone sarcomas after arthroplasty of large bones and joints. Voprosy kurortologii, fizioterapii, i lechebnoi fizicheskoi kultury, 2020; 97(3): 53-59.
- Lopresti, M. et al. Rehabilitation pathway after knee arthroplasty with mega prosthesis in osteosarcoma. Recenti Progressi in Medicina, 2015; 106(8): 385-392.

Study design:

- Nicholson, S. Femoral-tibial replacement for osteosarcoma. Nurs Times, 1988; 84(7): 34-37.

Conference proceeding without full text:

- Winter, C. et al. The impact of an intervention to increase physical activity in patients with a bone tumor. Pediatric Blood and Cancer, 2011; 57(5): 778-779.

Magazine article:

- Dulin, D. Cancer rehabilitation. Facilitating early rehabilitation in limb-salvage patients. Oncology nursing forum, 1989; 16(1): 105.
